# Potent in Vitro α-Glucosidase Inhibition of Secondary Metabolites Derived from *Dryopteris cycadina*

**DOI:** 10.3390/molecules24030427

**Published:** 2019-01-24

**Authors:** Surriya Amin, Barkat Ullah, Mumtaz Ali, Abdur Rauf, Haroon Khan, Eugenio Uriarte, Eduardo Sobarzo-Sánchez

**Affiliations:** 1 Department of Botany, Islamia College University, Peshawar 25000, Pakistan; saafridi2013@hotmail.com (S.A.); bu_barq@yahoo.com (B.U.); 2 Department of Chemistry, University of Malakand, Upper Dir 23050, Pakistan; mumtazali@uom.edu.pk; 3 Department of Chemistry, University of Swabi, Khyber Pakhtunkhwa, Anbar 23430, Pakistan; mashaljcs@yahoo.com; 4 Department of Pharmacy, Abdul Wali Khan University, Mardan 23200, Pakistan; 5 Instituto de Ciencias Químicas Aplicadas, Universidad Autónoma de Chile, Santiago 7500912, Chile; eugenio.uriarte@uautonoma.cl; 6 Departamento de Química Orgánica, Facultad de Farmacia, Universidad de Santiago de Compostela, 15782 Santiago de Compostela, Spain; eugenio.uriarte@usc.es; 7 Instituto de Investigación e Innovación en Salud, Facultad de Ciencias de la Salud, Universidad Central de Chile, Santiago 8330507, Chile; 8 Laboratory of Pharmaceutical Chemistry, Faculty of Pharmacy, University of Santiago de Compostela, 15782 Santiago de Compostela, Spain

**Keywords:** *Dryopteris cycadina*, isolated compounds, α-glucosidase inhibition, molecular docking

## Abstract

α-glucosidase is responsible for the hydrolysis of complex carbohydrates into simple absorbable glucose and causes postprandial hyperglycemia. α-glucosidase inhibition is thus the ideal target to prevent postprandial hyperglycemia. The present study was therefore designed to analyze the effects of various compounds isolated from *Dryopteris cycadina* against α-glucosidase including β-Sitosterol **1**, β-Sitosterol3-*O*-β-d-glucopyranoside **2**, 3, 5, 7-trihydroxy-2-(*p*-tolyl) chorman-4-one **3**, Quercetin-3-0-β-d-glucopyranoside (3^/^→0-3^///^)- β-d- Quercetin -3-0- β –d-galactopyranoside **4** and 5, 7, 4^/^-Trihydroxyflavon-3-glucopyranoid 5. The in vitro spectrophotometric method was used for the analysis of test compounds against possible inhibition. Similarly, molecular docking studies were performed using the MOE software. These compounds showed concentration-dependent inhibition on α-glucosidase, and compounds **1** (IC_50_: 143 ± 0.47 µM), **3** (IC_50_:133 ± 6.90 µM) and **5** (IC_50_: 146 ± 1.93 µM) were more potent than the standard drug, acarbose (IC_50_: 290 ± 0.54 µM). Computational studies of these compounds strongly supported the in vitro studies and showed strong binding receptor sensitivity. In short, the secondary metabolites isolated from *D. cycadina* demonstrated potent α-glucosidase inhibition that were supported by molecular docking with a high docking score.

## 1. Introduction

Diabetes is characterized by persistent endocrine disorder of multiple etiologies caused by a comparatively low or complete absence of insulin [1]. It is characterized by hyperglycemia [2] with the irregular metabolism of carbohydrates, proteins, fats, and electrolytes due to a lack of insulin or the insensitivity of target cells to insulin [3], which results the elevation of blood sugar, leading to hyperglycemia [4]. Hyperglycemia is the initial metabolic disorder of type 2 diabetes mellitus and if left untreated may develop into serious complications like damaged organs and systems, particularly the eyes, kidneys, nerves, blood and cardiovascular system [5,6]. The major defect that occurs in early diabetes is postprandial hyperglycemia, which mainly occurs due to excessive eating, obesity, age and lack of exercise [7,8,9]. α-glucosidase (EC 3.2.1.20) is a hydrolytic enzyme, mainly secreted by cells lining in the brush borders of epithelial cells of small intestine [10,11]. α-glucosidase mainly target α-1→4 glycosidic linkages and is responsible for the hydrolysis of complex carbohydrates into simple absorbable glucose. α-glucosidase belong to the sub-subclass hydrolases that cause the hydrolysis of various substances in the body [12,13]. In the 1970s, α-glucosidase hydrolases were discovered that were used to recover postprandial hyperglycemia, and which were officially approved as an antidiabetic drug later in the 1990s [14,15]. In this context, more potent and tolerable agents could account for the effective management of the disease.

*Dryopteris cycadina* is a medicinal plant from the Dryopteridaceae family. The *Dryopteris* genus consists of 250 species of ferns and are mostly distributed in the temperate northern hemisphere of Eastern Asia [16]. A literature survey revealed numerous medicinal uses of the *Dryopteris* species in folk medicine [17]. It is helpful in the treatment of rheumatism, epilepsy, pain [18], and as a remedy for snake bites and fungal infections [19], as well as diabetes management. The isolated compounds from this plant are flavonoid glycosides, which possess antioxidant, antibacterial and antitumor properties, and HIV-1 reverse transcriptase inhibitory activity [20,21]. The chemical constituents in *D. cycadina* includes steroids, phenols, phenolic glycosides, flavonoid glycosides, flavonoids, terpenoids, phenylpropranoids and others [22,23]. Kaempferol-3, 4^/^-di-O-α- l-rhamnopyranoside and Kaempferol-3,7-di-O-α- l-rhamnopyranoside isolated from the *D. cycadina* plant showed remarkable antinociceptive activity [24,25].

Based on the strong pharmacological, phytochemical and traditional uses of different species of *Dryopteris,* and it’s potential as an antidiabetic, the current study was designed to test compounds **1**–**5** isolated from *D. cycadina* against α- glucosidase for possible inhibition, and computational studies were carried out to test receptor binding sensitivity.

## 2. Results

### 2.1. Effect of In Vitro α-Glucosidase Activity

**Figure 1:**
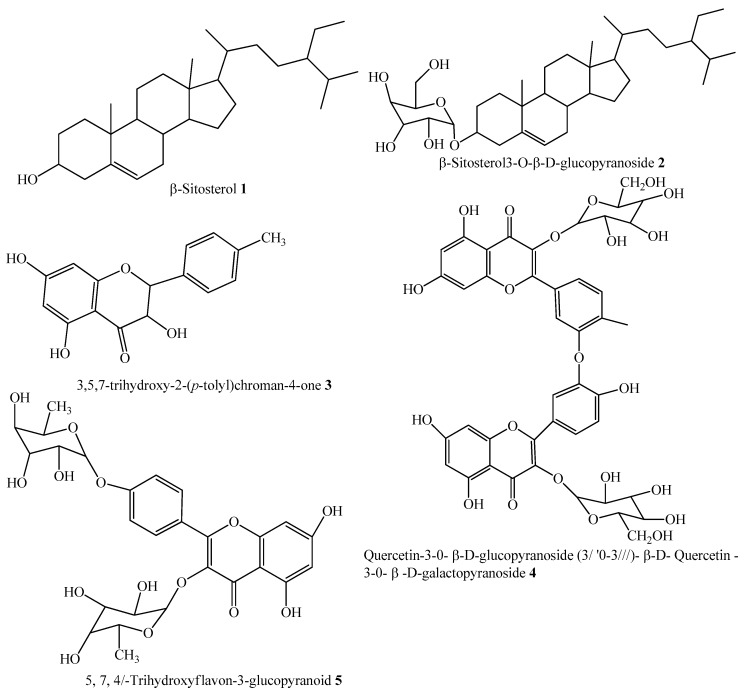
Structure of test compounds **1–5** isolated from *Dryopteris cycadina*.

**Figure 2:**
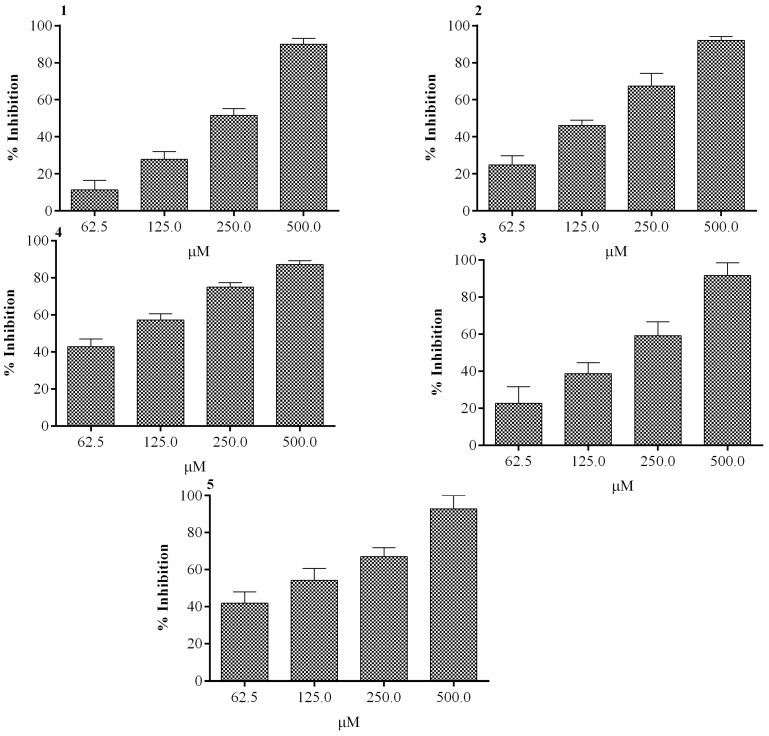
Per cent inhibition of test compounds **1**–**5** from *Dryopteris cycadina* against α-glucosidase at various concentrations. Values are expressed as mean ± SEM of three independent readings.

Compounds **1**–**5** isolated from *D. cycadina*, as seen in Figure 1, were screened for in vitro α-glucosidase inhibitory activity. Compound **1,** at a concentration of 500 μM, exhibited the maximum α-glucosidase inhibitory activity of 92.9% (Figure 2), while its half maximal inhibitory concentration was observed as 143 ± 0.47μM, as seen in Table 1. The maximum inhibition of compound **2** was 92% and was observed at 500 µM, as seen in Figure 2, with an IC_50_ value of 314 ± 4.58 μM, see Table 1. Compound **3** showed marked inhibitory activity against α-glucosidase at various concentrations, with a maximum inhibition of 94% at 500 µM, see Figure 2. The half maximal concentration (IC_50_) was calculated as 133 ± 6.90 µM, as shown in Table 1. The inhibitory activity showed by compound **4** against α-glucosidase was 87.2%, and was observed at a concentration of 500 µM, see Figure 2, and had an IC_50_ value of 298 ± 0.67µM, as seen in Table 1. Compound **5** demonstrated excellent inhibition of 97.1% at 500 µM, as seen in Figure 2, and its calculated IC_50_ was 146 ± 1.93 µM, see Table 1.

### 2.2. Effect of Molecular Docking

**Figure 3:**
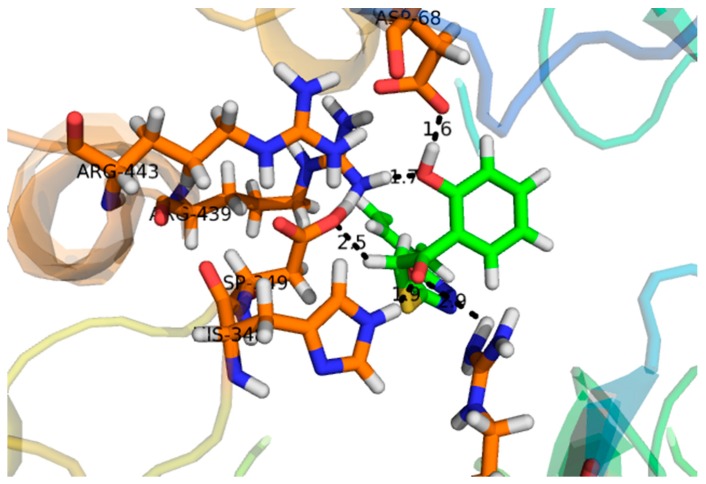
Represents the 3D structures of Compound **1** showing binding potential. Red color in the image shows position of oxygen, green benzene ring, white hydrogen atom, brown color indicates amino acid sequence and blue color indicate nitrogen or amino group.

**Figure 4:**
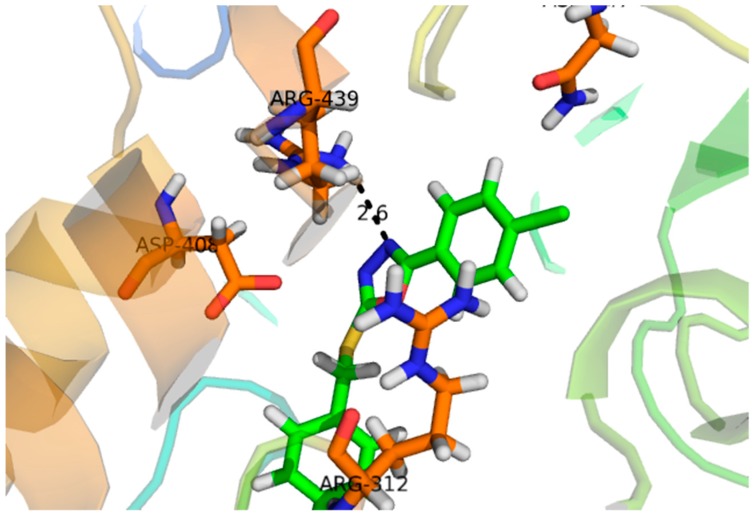
Represents the 3D structures of Compound **2** showing binding potential. Red color in the image shows position of oxygen, green benzene ring, white hydrogen atom, brown color indicates amino acid sequence and blue color indicate nitrogen or amino group.

**Figure 5:**
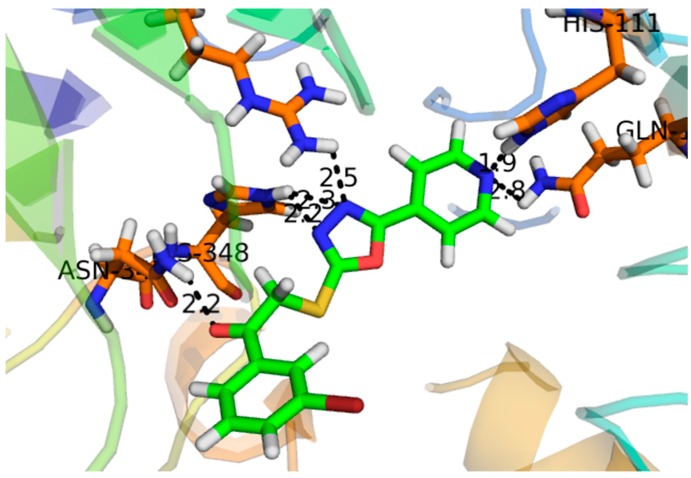
Represent the 3D structures of Compound **3** showing binding potential. Red color in the image shows position of oxygen, green benzene ring, white hydrogen atom, brown color indicates amino acid sequence and blue color indicate nitrogen or amino group.

**Figure 6:**
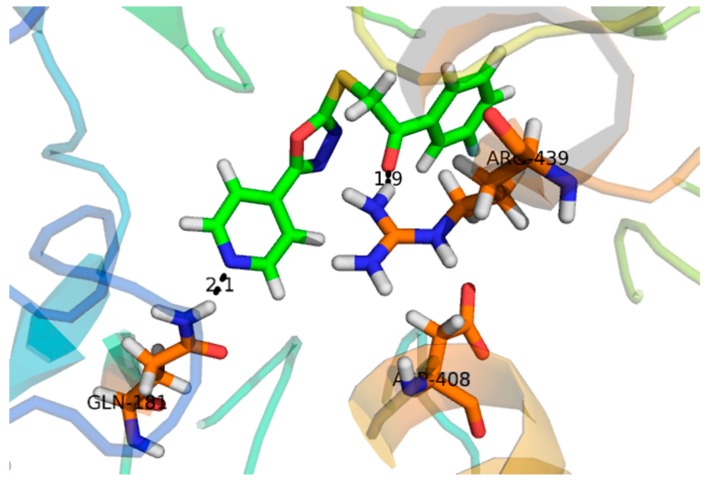
Represents the 3D structures of Compound **4** showing binding potential. Red color in the image shows position of oxygen, green benzene ring, white hydrogen atom, brown color indicates amino acid sequence and blue color indicate nitrogen or amino group.

**Figure 7:**
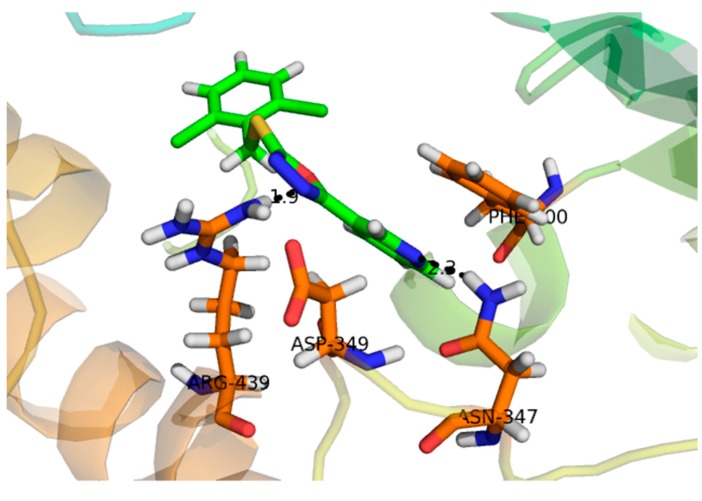
Represents the 3D structures of Compound **5** showing binding potential. Red color in the image shows position of oxygen, green benzene ring, white hydrogen atom, brown color indicates amino acid sequence and blue color indicate nitrogen or amino group.

Compound **1**, with a docking score of −16.097 (Table 2) displayed significant hydrogen bonding interactions with the four catalytic residues of Arg-442, Asp-215, Asp-352 and Gln-182 of the receptor α-glucosidase, as seen in Figure 3. Compound **2**, with a docking score of −7.756, as seen in Table 2, showed one binding interaction with α-glucosidase. The hydrogen binding interaction was found with amino acid Asn-415 of α-glucosidase, see Figure 4. One arene–arene and eight hydrogen binding interactions were observed in compound **3** with a docking score of −22.480. The active site residues such as Arg-315, Asp-307, His- 280, Lys-156, Ser-240 and Thr-310 indicated hydrogen binding interactions, while Tyr-158 exhibited an arene–arene interaction with α-glucosidase, as seen in Figure 5. The docking score of compound **4** was −12.931, indicating that two different interactions were found, that is, one hydrogen bond and other arene–arene interaction with the active site residue Arg-442 and Tyr 158, as seen in Figure 6. Compound **5**, with a docking score of −15.752, had one arene–arene and three hydrogen binding interactions—Asp-242, Lys-156, Pro-312 and Tyr-158—with the active site residue of the receptor (Figure 7).

## 3. Materials and Methods

### 3.1. Materials

α-glucosidase (EC3.2.1.20) was obtained from Sigma Aldrich, and acarbose was obtained from Bayer, Pakistan. An ELISA Micro Plate Reader (Emax) from Molecular Devices and isolated compounds **1**–**5** from *D. cycadina* were used.

### 3.2. Assay Protocol

The α-glucosidase (*Saccharomyces cerevisiae*) inhibitory assay was carried out with slight modifications according to the method described in [21,26,27]. For the evaluation of α-glucosidase inhibitory activity, 10 µl of freshly prepared phosphate buffer (pH 6.8) were plated in triplicate for a micro-well plate with the help of a micropipette. Then, 30 µL of α-glucosidase enzyme with a concentration of 0.017 units/mL of 70% ethanol and 10 µL of test compound solution were added to the same well plate. The plates were then incubated for 15 min at 37 °C and an initial reading was taken at 405 nm using the ELISA micro plate reader. Furthermore, 100 µl of 0.7mM PNPG substrate was added to each well of the plate and again incubated at 37 °C for 30 min, forming a yellow color. The final reading was taken again at λ _max_ 405 nm using the ELISA plate reader. A total of 70% ethanol was used as a negative control, whereas acarbose was used as a positive control. Each reading was taken in triplicate. The percent inhibition was calculated using the following formula, % inhibition = [(*A* negative control − *A* test sample)/*A* negative control] × 100, where A is absorbance.

### 3.3. Half-Maximal Inhibitory Concentration of Compounds (IC_50_)

The compound that exhibited a 50% or greater inhibition on α-glucosidase was subjected to IC_50_ determination. The half-maximal inhibitory concentration (IC_50_) of the active compounds was determined by preparing various amounts of test solution—like 500 µM, 250 µM, 125 µM and 62.5 µM—and their inhibitory studies were determined using the method described earlier. The half-maximal inhibitory concentration values were determined using the Graphpad Prism version 7.0 software (San Diego, CA, USA. All values are represented as mean ± SEM.

### 3.4. Computational Study

The three-dimensional structure for α-glucosidase of *Saccharomyces cerevisiae* has not yet been solved. Thus, the three-dimensional structure of α-glucosidase was generated using the Molecular Operating Environment (MOE 2010.11) software and the molecular docking study was performed on the same software. The MOE-Dock was used as the docking software implemented in MOE and ligplot was implemented in MOE for the purpose of visualizing the interaction between protein and ligand. The primary sequence of the α glucosidase was retrieved using Uniprot (Universal Protein Resource) (http://www.uniprot.org/) in Federal Acquisition S Streamlining (FASTA) format and the target sequence was then kept in the text-file for further evaluation [28]. The accession number of α glucosidase of *Saccharomyces cerevisiae* was P07265. Then Protein-BLAST was performed to identify homologs in the PDB (RCSB Protein Databank) [9,29,30]. Hence, the crystal structure of *Saccharomyces cerevisiae* (PDB Id: 3A47_A), which has 72% sequence identity to the target protein, was selected as the template for the target protein sequence for the prediction of the tertiary structure of the target protein. The amino acid sequence of the target protein in FASTA format was copied and pasted into the sequence editor of the MOE software. Then the template protein was loaded into the same MOE software. Prior to docking, the 2D structures of all inhibitors were drawn using the Cambridge Soft Chem3D Ultra Version 10.0 by Cambridge Soft Corp, MA, USA. Protein-ligand docking studies were performed using the MOE 2009.10 software package. Ligands were optimized using the default parameters of the MOE-DOCK software, including energy minimization, protonation and the removal of nonpolar hydrogens. Now the entire ligand database was docked into the binding pocket of the protein using the triangular matching docking method. Ten different conformations of each ligand–protein complex was generated, each possessing its specific docking score. The docking process was repeated for the validation of the docking method for the type of interaction. Finally, the two- and three-dimensional images of each complex were analyzed and taken.

### 3.5. Statistical Analysis

All the data are expressed as the mean ± SEM of three independent readings. The IC_50_ values were calculated using the Graph Pad Prism version 7.0 software (San Diego, CA, USA), while the docking studies were performed using the MOE (2009-10) software.

## 4. Discussion

The present study revealed a significant in vitro α-glucosidase assay that was strongly complimented by computational studies. In vitro α-glucosidase assay is a simple, economical and time-saving method for the analysis of the inhibitory potential of various compounds. In the present study, the inhibitory activity of isolated bioactive constituents was determined by measuring its spectrophotometric absorbance at a wavelength of 405 nm using ELISA. Besides this, the color change of the reaction mixture from deep yellow to light yellow also indicated significant inhibition of the α-glucosidase [30,31].

Compound **3** was found to be the most active compound, with an IC_50_ value of 133 ± 6.90 µM, and thus elicited marked inhibitory activity against α-glucosidase. It could be attributed to π-electrons of the hydroxyphenyl ring, and the hydrogen bonding interactions of the two pyran rings were engaged to create strong interactions with the amino acids of the α-glucosidase. Compound **1** exhibited a significant inhibitory effect, with an IC_50_ value of 143 ± 0.47 µM. However, previous studies showed it to be inactive [32]. Compound **1,** which had a docking score of -16.097, exhibited excellent binding interactions with the active residues of the amino acids. The activity of this compound may be attributed to the interaction of the hydroxyl group of the tetrahydropyran group with the active sites of the enzyme, such as Arg-442, Asp-215, Asp-352 and Gln-182, as seen in Figure 3. It is assumed that the strong inhibitory activity of this compound may be attributed to the interaction of the hydroxyl group of the tetrahydropyran group with the active sites of the enzyme. Compound **5**, with an IC_50_ value of 146 ± 1.93 µM, exhibited marked inhibitory activity against α-glucosidase due to the hydrogen bonding interaction of the pyran ring. Moreover, thee chromen ring also showed two hydrogen bonding interactions and a π-electron interaction with the receptor atoms. Compound **4** and **2** showed lesser α-glucosidase inhibitory activity, with an IC_50_ value of 298 ± 0.67 and 314 ± 4.58 µM, lower than the standard acarbose (290 ± 0.54 µM), respectively. In compound **2**, the weak inhibitory activity was due to the single bond interaction of the oxygen atom of the phenanthrene ring with the receptor atoms, while compound **5** exhibited weak α-glucosidase inhibitory activity due to weak bonding interactions of the chromen ring with receptor atoms.

The Molecular Operating Environment (MOE) docking program (Molecular operating environment MOE. 2008. C.C.G.I.M, Quebec Canada, MOE-Dock, Chemical Computing Group. 1998. Inc., Montreal Quebec Canada), was used to examine the binding modes of the test compounds against the α-glucosidase enzyme. The binding modes of all test compounds **(1**–**5)** isolated from *D. cycadina* against α-glucosidase enzyme was examined by molecular docking studies, and the results are provided in Table 2. The docking studies illustrated that the order of bioactivity of the tested compounds followed the same trend as was observed for the IC_50_ values. All the compounds interacted chemically with the active site as well as catalytic residues of the α-glucosidase. Based on the docking results, compounds **1, 3** and **5** were considered to be the most active compounds, as seen in Table 2. The most active conformer for each compound was selected based on the **(S)** score from the docking results. A lower **(S)** score indicates a stable pose and good interactions [33]. The order of activity of these compounds was 3 > 1 > 5 > 4 > 2, which follows the same trend as the in vitro biological assay, seen in Table 1. 

Compound **3** (IC_50_ 136 ± 1.10 µM) −22.480 acted at different interaction sites with α-glucosidase amino acid residues. The residue Tyr-158 showed arene–cation interactions with π-electrons of the hydroxyphenyl ring. Moreover, the amino acid residues Arg-315, Asp-307, His- 280, Lys-156, Ser-240 and Thr-310 of the two pyran rings were engaged in making strong hydrogen binding interactions, as seen in Figure 5. Compound **5** (IC_50_ 143 ± 0.47 µM) −15.752 interacted with the α-glucosidase residue i.e., Pro-312 by a hydrogen bonding interaction of the pyran ring. Moreover, the chromen ring also showed two hydrogen bonding interactions and a π-electron interaction with receptor atoms, such as Asp-242, Lys-156 and Tyr-158, as seen in Figure 7. Compounds **4** (IC_50_ 298 ± 0.67 µM) and **2** (IC_50_ 314 ± 4.58 µM) showed poor α-glucosidase interactions with amino acid residues of α-glucosidase, for example compound **4** −12.931 exhibited a weak interaction with the chromen ring and the active site residue Arg-442 and Tyr 158, whereas compound **2** −7.756 exhibited poor α-glucosidase inhibitory activity and had a single interaction of the phenanthrene ring with Asn-415 of the active site of the enzyme, as seen in Figure 4. Due to the limited quantity of compounds 1–5, kinetic studies were not performed. It is suggested that kinetic studies are conducted in the future to determine the mechanism of these compounds.

## 5. Conclusions

In short, over the years, natural products have shown outstanding therapeutic potential [34,35,36,37,38,39]. In our study, compounds **1**–**5** isolated from *D. cycadina* possessed strong α-glucosidase inhibition when studied at different concentrations with an overall, concentration-dependent effect. These compounds elicited marked potency in terms of the IC_50_ values and were better than the standard drug used, acarbose. Similarly, in molecular docking studies these compounds exhibited strong binding potentials, which supported the in vitro experimental findings. In this context, further detailed studies are recommended to explore the mechanisms, safety and clinical aspects of these molecules.

## Figures and Tables

**Table 1:** Half-maximal inhibitory concentrations of test compounds (**1**–**5**) isolated from *Dryopteris cycadina.*

α- Glucosidase	**Compounds**	**IC_50_ ± SEM (µM)**
	**1**	143 ± 0.47
	**2**	314 ± 4.58
	**3**	133 ± 6.90
	**4**	298 ± 0.67
	**5**	146 ± 1.93
	Acarbose	290 ± 0.54

Values are expressed as mean ± SEM of three independent readings.

**Table 2:** Molecular docking score of the test compounds with interacting amino acids of the active site of α-glucosidase enzyme.

Compound	Docking Score	Interacting Residues of the Receptor
**1**	−16.097	ASP-215, ASP-352, ARG-442, GLN-182
**2**	−7.756	ASN-415
**3**	−22.480	ARG-315, ASP-307, HIS-280, LYS-156, SER-240, THR-310, TYR-158
**4**	−12.931	ARG-442, TYR-158
**5**	−15.752	ASP-242, LYS-156, PRO-312, TYR-158

Docking results of test compounds (**1**–**5**) isolated from *Dryopteris cycadina* against α-glucosidase.
